# NLRP3 promotes tumor growth and metastasis in human oral squamous cell carcinoma

**DOI:** 10.1186/s12885-018-4403-9

**Published:** 2018-05-02

**Authors:** Han Wang, Qingqiong Luo, Xiaodong Feng, Ruiyang Zhang, Jiang Li, Fuxiang Chen

**Affiliations:** 1 Department of Clinical Immunology, Ninth People’s Hospital, Shanghai Jiao Tong University School of Medicine, 639 Zhizaoju Road, Shanghai, 200011 China; 2 Department of Oral Pathology, Ninth People’s Hospital, Shanghai Jiao Tong University School of Medicine, Shanghai, 200011 China

**Keywords:** NLRP3 inflammasome, Oral squamous cell carcinoma, Proliferation, Migration, Invasion

## Abstract

**Background:**

Inflammasomes are reported to be abnormally expressed and activated in several malignancies and play important roles in tumor development. The present study was designed to investigate the expression and function of the NLR family pyrin domain containing protein 3 (NLRP3) inflammasome in oral squamous cell carcinoma (OSCC).

**Methods:**

NLRP3 expression in OSCC cell lines and the normal human immortalized oral epithelial cells (HIOEC) was determined by real-time PCR and western blot. Immunohistochemistry was used to examine the expression of NLRP3 and IL-1β in the paraffin-embedded OSCC tissues. The proliferation of OSCC cells was detected by the 3-(4,5-dimethylthiazol- 2-yl)-2,5-diphenyl tetrazolium bromide (MTT) assay and cell colony formation ability of the OSCC cells was also evaluated. Tumor cell migration or invasion was measured by the transwell assay and related protein markers were determined by western blot. A mouse xenograft model was established to investigate the OSCC tumor growth in vivo.

**Results:**

Significant higher expression of NLRP3 was observed in the OSCC cells. Obvious expression of NLRP3 and IL-1β was found in the paraffin-embedded OSCC tissues, and the NLRP3 expression levels were correlated with the tumor size, lymphonode metastatic status and IL-1β expression. Downregulating NLRP3 expression markedly reduced the cleavage of caspase-1 and production of IL-1β in OSCC cells. NLRP3 knockdown also inhibited the proliferation, migration and invasion of OSCC cells. Further investigation indicated that expressions of E-cadherin and vimentin in OSCC cells were increased, while N-cadherin expression was decreased after NLRP3 knockdown. Downregulating NLRP3 expression in OSCC cells significantly reduced the tumor growth in vivo.

**Conclusions:**

Our data suggested that the increased expression of NLRP3 in OSCC was associated with tumor growth and metastasis. NLRP3 may be considered as a potential target for OSCC therapy.

**Electronic supplementary material:**

The online version of this article (10.1186/s12885-018-4403-9) contains supplementary material, which is available to authorized users.

## Background

Oral squamous cell carcinoma (OSCC) is the most common type of cancer in oral cavity, which accounts for more than 90% of all oral cancers [1]. Approximately 300,000 new cases of OSCC patients occurs worldwide annually [2]. Although there are much improvements in multimodality therapy including surgery, radiation and chemotherapy, the overall survival rate for OSCC still ranges from 50 to 60% [3, 4]. Therefore, it is important to investigate the molecular mechanisms of the occurrence and development of OSCC, which may ultimately help the exploration and establishment of more effective therapeutic strategies.

Chronic inflammation has been reported to play critical roles in carcinogenesis and tumor progression [5–8] and cancer-related inflammation has been recognized as the “seventh hallmark of cancer” [9]. Recently, inflammasomes-mediated inflammation in cancer has aroused great interests in the field [10, 11]. Inflammasomes are large protein complexes typically consisting of the nucleotide-binding and oligomerization domain (NOD)-like receptor (NLR), adapter protein apoptosis-associated speck-like protein containing CARD (ASC) and caspase-1 [12, 13]. Once activated by a diverse range of “danger signals” like oxidative stress, pathogens, metabolic and tissue damage products etc., the inflammasome complex subsequently cleaves pro-IL-1β to its mature bioactive form via the activated caspase-1 [14, 15]. Studies of Fujita et al. reported that the constitutively active NLR pyrin domain-containing protein 3 (NLRP3) inflammasome in human melanoma cells mediated autoinflammation via caspase-1 processing and IL-1β secretion [16] and NLRP1 inflammasome activation promoted tumor growth in metastatic melanoma [17]. Normand et al. demonstrated the NLRP6 inflammasome controlled epithelial self-renewal and colorectal carcinogenesis upon injury [18]. Kolb et al. indicated obesity associated NLR family CARD domain-containing protein 4 (NLRC4) inflammasome activation drove breast cancer progression [19]. Other investigations have involved inflammasomes in bladder cancer [20], gastric cancer [21], and leukemia [22]. However, the roles of inflammasomes, particularly NLRP3 inflammasome in OSCC have not been fully elucidated.

Thus we performed the present study and found that NLRP3 was aberrantly overexpressed in OSCC cells and tissues. The NLRP3 expression in OSCC tissues was positively correlated with tumor size, lymphonode metastasis status and IL-1β expression (product of the activated NLRP3 inflammsome). Moreover, knockdown of NLRP3 inhibited OSCC cell proliferation, migration and invasion. In the xenograft mouse model, we further demonstrated that suppressing NLRP3 expression could reduce the growth of OSCC in vivo.

## Methods

### Patients and tissue specimens

Seventy-seven primary OSCC tumor samples were collected from patients of Shanghai Ninth People’s Hospital at the Department of Oral and Maxillofacial–Head and Neck Oncology from January 2010 to July 2011, and all the patients had no previous chemotherapy or radiotherapy. The pathological grades were evaluated according to the standard of American Joint Committee on Cancer (AJCC) and all study protocols were approved by the Ethics Committee of the hospital. The clinical parameters of the OSCC patients were included in Table 1.

**Table 1:** Correlation between NLRP3 expression and clinicopathological features, IL-1β expression in patients with OSCC (*n* = 77)

Characteristics	n(77)	NLRP3 expression			*P*-value^a^
		negative (20)	weak(24)	positive (33)	
		n(%)	n(%)	n(%)	
Age(years)					0.436
≤ 60	40	10 (25)	15 (37.5)	15 (37.5)	
>60	37	10 (27)	9 (24.3)	18 (48.7)	
Sex					0.718
Male	57	15 (26.3)	19 (33.3)	23 (40.4)	
Female	20	5 (25)	5 (25)	10 (50)	
Smoking status^b^					0.491
Current/former	36	8 (22.2)	10 (27.8)	18 (50)	
Never	41	12 (29.3)	14 (34.1)	15 (36.6)	
Alcohol use^c^					0.561
Yes	26	5 (19.2)	8 (30.8)	13 (50)	
No	51	15 (29.4)	16 (31.4)	20 (39.2)	
Location					0.403
Gingiva	23	4 (17.4)	6 (26.1)	13 (56.5)	
Tongue	31	11 (35.5)	11 (35.5)	9 (29)	
Floor of mouth	10	1 (10)	4 (40)	5 (50)	
Buccal	13	4 (30.7)	3 (23.1)	6 (46.2)	
AJCC stage					0.018*
I	21	10 (47.6)	8 (38.1)	3(14.3)	
II	41	8 (19.5)	11(26.8)	22 (53.7)	
III	15	2 (13.3)	5 (33.3)	8 (53.4)	
T stage					0.015*
T1	9	5 (55.6)	3 (33.3)	1 (11.1)	
T2	24	10 (43.5)	6 (26.1)	8(30.4)	
T3	28	3 (10.7)	10(35.7)	15 (53.6)	
T4	16	2 (12.5)	5 (31.2)	9(56.3)	
N stage					0.008**
N0	39	15(38.5)	15 (38.5)	9 (23)	
N1	23	3 (13)	6 (26.1)	14 (60.9)	
N2	15	2 (13.3)	3 (20)	10 (66.7)	
IL-1β expression					0.004**
Negative	21	12(57.2)	4(19)	5(23.8)	
Weak	21	4(19)	7(33.3)	10(47.7)	
Positive	35	4(11.4)	13(37.1)	18(51.5)	

^a^*P*-values are based on chi-squared or Fisher’s exact test. *P* < 0.05 indicates a significant association among the variables. (*P<0.05, **P<0.01)

^b^Former/current smokers defined as at least a one pack-year history of smoking

^c^Alcohol intake was defined as current alcohol intake of more than one drink per day for 1 year. Others were classified as no alcohol intake

### Animals

Male BALB/c nude mice about four-week-old were purchased from Shanghai Laboratory Animal Center (Shanghai, China) and kept in animal care facilities under pathogen-free conditions. The procedures of animal experiments were conducted in line with the institutional guidelines for the use of laboratory animals (The Ministry of Science and Technology of China, 2006) and also had approval of the Ethics Committee of the hospital.

### Cell lines

The human immortalized oral epithelial cells (HIOEC) and three OSCC cell lines- WSU-HN4, WSU-HN6 and CAL27 were kindly provided by the Key Laboratory of Stomatology, Shanghai Ninth People’s Hospital. The OSCC cells were cultured in Dulbecco’s modified Eagle medium (DMEM) (Invitrogen, Carlsbad, CA, USA) supplemented with 1% penicillin-streptomycin and 10% fetal bovine serum (FBS) (Gibco, New York, NY, USA). HIOEC cells were cultured in defined Keratinocyte-SFM (Gibco). All cells were incubated in a humidified atmosphere containing 5% CO_2_ at 37 °C.

### Immunohistochemistry (IHC) staining

For IHC staining, the procedures were as described previously [23]. After deparaffinating and rehydrating, the 5 μm thick tumor tissue sections were heated by water bath with citric acid buffer for 20 min and blocked in 5% normal goat serum for 30 min, and then incubated with either anti-NLRP3 antibody (1:400, sigma, St Louis, MO, USA) or anti-IL-1β antibody (1:200, Abcam, Cambridge, UK) at 4 °C overnight. Subsequently, the GTVision Two-step Visualization System (Genetech, Shanghai, China) was used to visualize the immunostaining and hematoxylin was used to counter-stain. Finally, the sections were examined under light microscopy and evaluated by the Immuno-Reactive-Score (IRS), which was calculated by multiplying staining proportion score (PS) and staining intensity score (IS). PS was defined as 0 (0%), 1 (1%~ 25%), 2 (26%~ 50%), 3 (51%~ 75%), and 4 (76%~ 100%). IS was classified as 0 (negative), 1 (weak), 2 (moderate) and 3 (strong). According to the obtained IRS scores, patients were divided into three groups: negative expression group with IRS = 0, weak expression group with IRS = 1~ 3 and strong positive expression group with IRS = 4~ 12.

### RNA extraction and real-time PCR

To determine the mRNA expression of NLRP3, total RNA in HIOEC and OSCC cells was extracted using the TRIzol reagent (Invitrogen, San Diego, CA, USA) and real-time PCR were carried out as described previously [23]. The PrimeScript RT Reagent kit (TaKaRa, Shiga, Japan) was adopted for reverse transcription. Real-time PCR was carried out using the SYBR Premix Ex Taq II (TaKaRa) reaction system and cycling conditions were set according to the manufacturer’s instructions. GAPDH was used as an endogenous control to normalize differences in the amount of total RNA of different samples. Primer sequences were as follows. NLRP3: sense 5′-CCATCGGCAAGACCAAGA-3′, anti-sense 5′-ACAGGCTCAGAATGCTCAT C-3′. GAPDH: sense 5′-TGACTTCAACAGCGACACCCA-3′, anti-sense 5′-CAC CCTGTTGCTGTAGCCAAA-3′.

### Western blot analysis

Cells were lysed in Radio Immunoprecipitation Assay (RIPA) buffer (Beyotime Biotechnology, Nanjing, China) and total proteins were denatured after quantification. Same amount of protein was loaded on sodium dodecyl sulfate (SDS) polyacrylamide gels for electrophoresis and then electrophoretically transferred onto polyvinylidene difluoride (PVDF) membranes (Bio-Rad, Hercules, CA, USA). Non-fat milk powder in Tris-buffered saline (TBS)/Tween20 (TBS/T) with a final concentration of 5% was used to block the non-specific sites. The blots were then incubated with various primary antibodies at 4 °C overnight. Fluorescent-based anti-rabbit or anti-mouse IgG secondary antibody (Fermentas, Vilnius, Lithuania) was used to detect the captured primary antibodies on the membranes. Finally, immunoreactive bands were analyzed by the Odyssey Infrared Imaging System (LI-COR Biosciences, Lincoln, NE, USA).

### Gene knockdown

To knockdown the expression of NLRP3 in OSCC cells, short hairpin RNA (shRNA) were constructed and purchased (Genechem, Shanghai, China) [23]. Sequences of the shRNA duplex targeting NLRP3 (shNLRP3) were: sense 5’-GATCCAGCCAA CAGGAGAACTTTCCTTCCTGTCAGAGAAAGTTCTCCTGTTGGCTTTTTTG-3′, anti-sense 5’-AATTCAAAAAAGCCAACAGGAGAACTTTCTCTGACAGGAAGG AAAGTTCTCCTGTTGGCTG-3′. The negative control (mock) vector was constructed with a sequence of 5’-GAAGCAGCACGACTTCTTC-3′. Twelve hours after lentivirus particles transfection, the cells were treated with puromycin for another 7 days to select the stable transfected cells. Real-time PCR and western blot were used to confirm the efficiency of gene knockdown.

### Cell proliferation and colony formation assay

Cells with different densities were planked into 96-well or 6-well plates and cultured for different time. To determine the cell proliferation, 3-(4,5-dimethylthiazol-2-yl)-2,5-diphenyl tetrazolium bromide (MTT, Sigma) solution was added into each well. After another 4 h incubation, supernatants were discarded and dimethyl sulfoxide (DMSO, Sigma) was supplemented to dissolve the formazan crystals. Finally, absorbance at wavelength of 490 nm was determined on a microplate reader (Bio-Rad). For colony formation assay, cell clones were stained with a solution containing 0.5% crystal violet and 25% methanol after three weeks’ culture. Excess dye was removed by rinses with tap water. Colonies (> 50 cells) were counted under a microscopy.

### Migration and invasion assays

The migration and invasion assays were performed in 8 μm Transwell Inserts (Corning, New York, USA) for 24 well plates with uncoated membranes or Matrigel (BD Biosciences, San Jose, USA) coated membranes, respectively. Briefly, OSCC cells (2 × 10^4^ cells) prepared in DMEM medium were loaded in the upper well, and complete culture medium supplemented with FBS was placed in the lower wells as chemo-attractant. After 12-h or 24-h incubation, the cells that did not penetrate the membrane on the upper chamber were gently removed with a cotton swab. Cells passed through the membrane were fixed and stained with 0.1% crystal violet solution. The inserts were thoroughly rinsed until the water became clear. Finally, cells numbers in the insert were estimated in 6 random fields of views under an inverted microscope.

### Establishment of OSCC xenograft mouse model

A total of 2 × 10^6^ WSU-HN6 cells with or without NLRP3 knockdown were injected subcutaneously into the back next to the right hind limb of the nude mouse and permitted to grow until palpable. Then tumors were measured every 4 days with a vernier caliper and tumor volumes were calculated according to the following formula: tumor volume (mm^3^) = A× B^2^ × 0.52. A is the longest diameter and B is the shortest diameter. Tumor-bearing mice were sacrificed at the end of the experiment. After being separated from the surrounding muscles and dermis, tumors were weighed or fixed with 4% phosphate-buffered paraformaldehyde.

### Statistical analysis

All the presented data and results were confirmed by at least three independent experiments. Data are shown as mean ± SD. The two-tailed Student’s *t* test was used to determine the statistical significance of the differences between two groups. The *χ*^*2*^ test was used to evaluate the relation between NLRP3 expression and different clinicopathological parameters of the patients, and the Fisher’s exact test was used to analyze the relation of NLRP3 expression and IL-1β expression. *P* < 0.05 was considered statistically significant. All statistical analyses were performed using the SPSS version 19.0 software (SPSS Inc., New York, NY, USA).

## Results

### NLRP3 expression is increased in OSCC cells

**Fig. 1:**
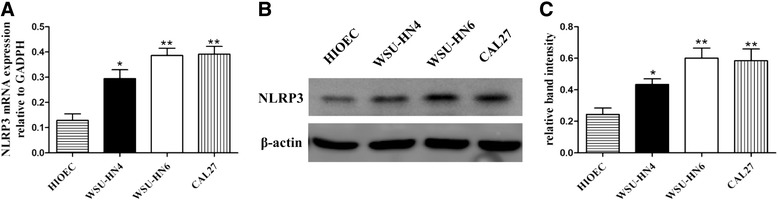
NLRP3 expression in cell lines. **a** Real-time PCR for NLRP3 mRNA determination. **b** Western blot analysis for NLRP3 protein detection. **c** The expression ratio of NLRP3 protein was quantified against β-actin. Results are representative of three experiments. (*P < 0.05, **P < 0.01)

To determine the expression levels of NLRP3 in OSCC cells, RT-PCR and western blot analysis were performed. Results indicated that the mRNA expression of NLRP3 was significantly higher in all three OSCC cell lines than that in HIOEC (Fig. 1a). Western blot analysis confirmed the higher expression of NLRP3 in OSCC cells (Fig. 1b and c). Thus, the aberrant overexpression of NLRP3 at the transcriptional and translational levels suggests that NLRP3 may be functionally important in OSCC.

### NLRP3 expression is associated with the clinicopathological characteristics of OSCC patients

**Fig. 2:**
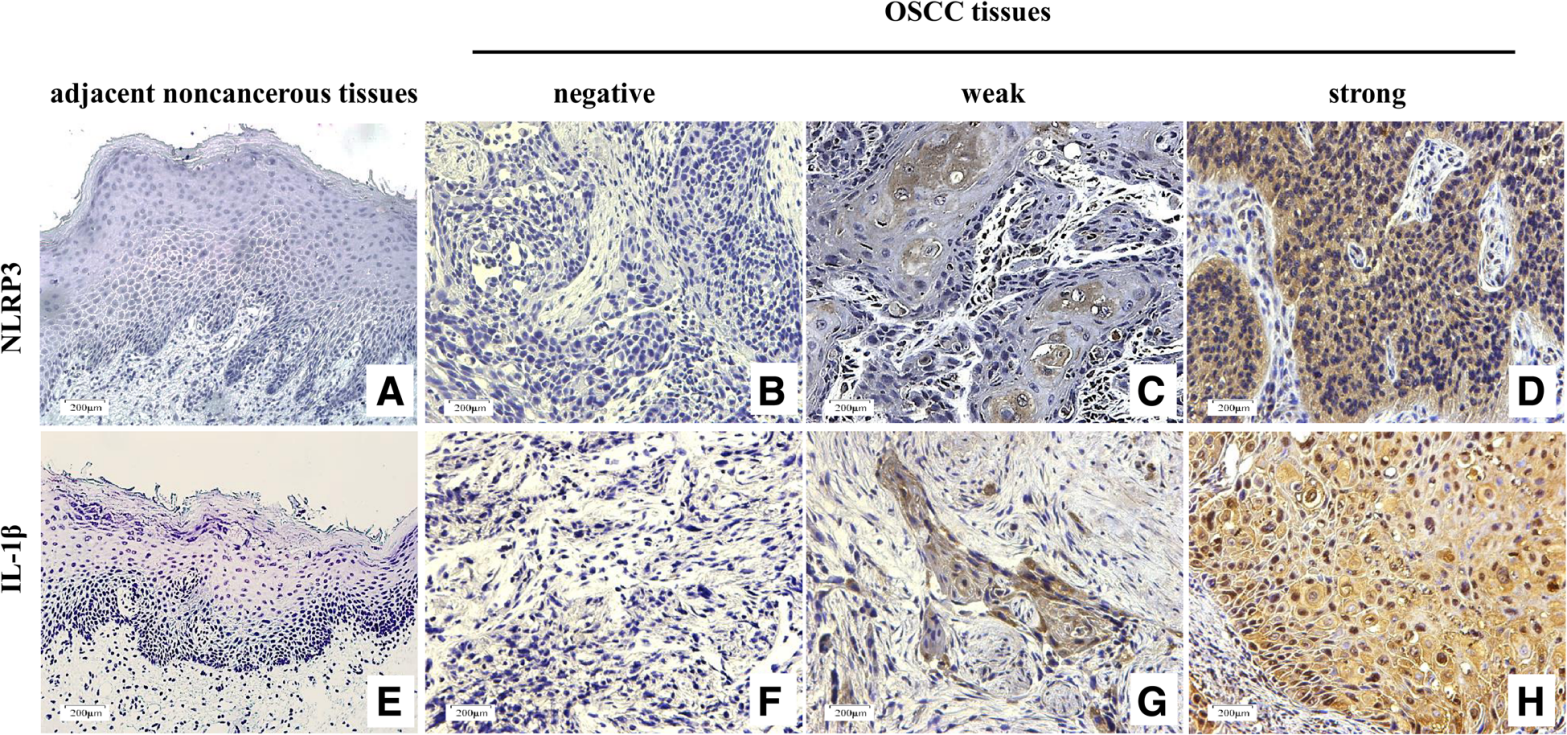
NLRP3 and IL-1β expression in tissues. NLRP3 (**a**-**d**) and IL-1β (**e**-**h**) expression in tissues were determined by IHC staining. (magnification 100× for adjacent noncancerous tissues and 200× for OSCC tissues)

To further identify the expression of NLRP3 in tissues, IHC staining was performed in 77 OSCC specimens. The staining score was calculated by multiplying staining proportion score (PS) and staining intensity score (IS). PS was classified as 0 (0%), 1 (1%~ 25%), 2 (26%~ 50%), 3 (51%~ 75%), and 4 (76%~ 100%). IS was classified as 0 (negative), 1 (weak), 2 (moderate) and 3 (strong). Patients with different expression were divided into three groups: negative expression group (IRS = 0), weak expression group (IRS = 1~ 3) and strong positive expression group (IRS = 4~ 12). In the OSCC tissues, positive staining of NLRP3 was observed in 74.03% (57/77) of the cases, with 42.11% (24/57) of weak expression and 57.89% (33/57) of strong expression (Fig. 2b-d and Additional file 1: Table S1). No obvious NLRP3 expression was found in the paired adjacent noncancerous tissues (Fig. 2a). The upregulated expression of NLRP3 was significantly associated with the AJCC stage (*P* = 0.018), T stage (*P* = 0.015) and N stage (*P* = 0.008) of OSCC (Table 1). As the activation of NLRP3 inflammasome leads to the maturation cleavage of pro-IL-1β, we evaluated IL-1β expression simultaneously. Similar expression patterns to NLRP3 were found for IL-1β (Fig. 2e-h), and a positive correlation between the NLRP3 and IL-1β was identified by the Fisher’s exact test (*P* = 0.004) (Table 1). Therefore, these data confirm the functional overexpression of NLRP3 in OSCC.

### NLRP3 knockdown inhibits the proliferation of OSCC cells

**Fig. 3:**
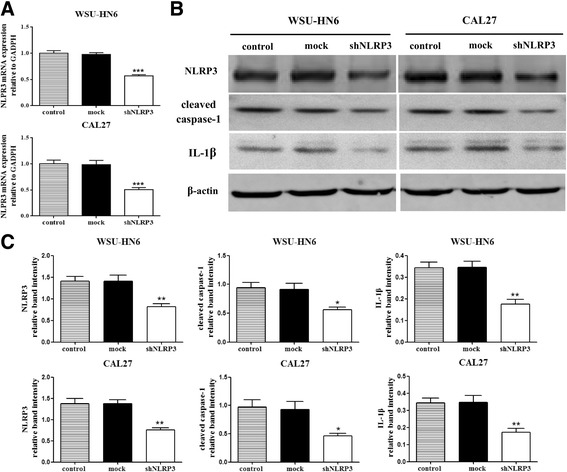
The identification of shRNA interference efficiency. WSU-HN6 and CAL27 cells were transfected with shNLRP3 or mock vectors. Control cells received no transfection. **a** Total mRNA was obtained 12 h after transfection. The mRNA expression level of NLRP3 was determined by real-time PCR analysis. **b** Total proteins were obtained 7 days after transfection as described in methods. Expression levels of NLRP3, cleaved caspase-1 and IL-1β were determined by western blot. **c** The expression ratio of the proteins was quantified against β-actin. Results are representative of three experiments. (*P < 0.05, **P < 0.01, ***P < 0.001)

**Fig. 4:**
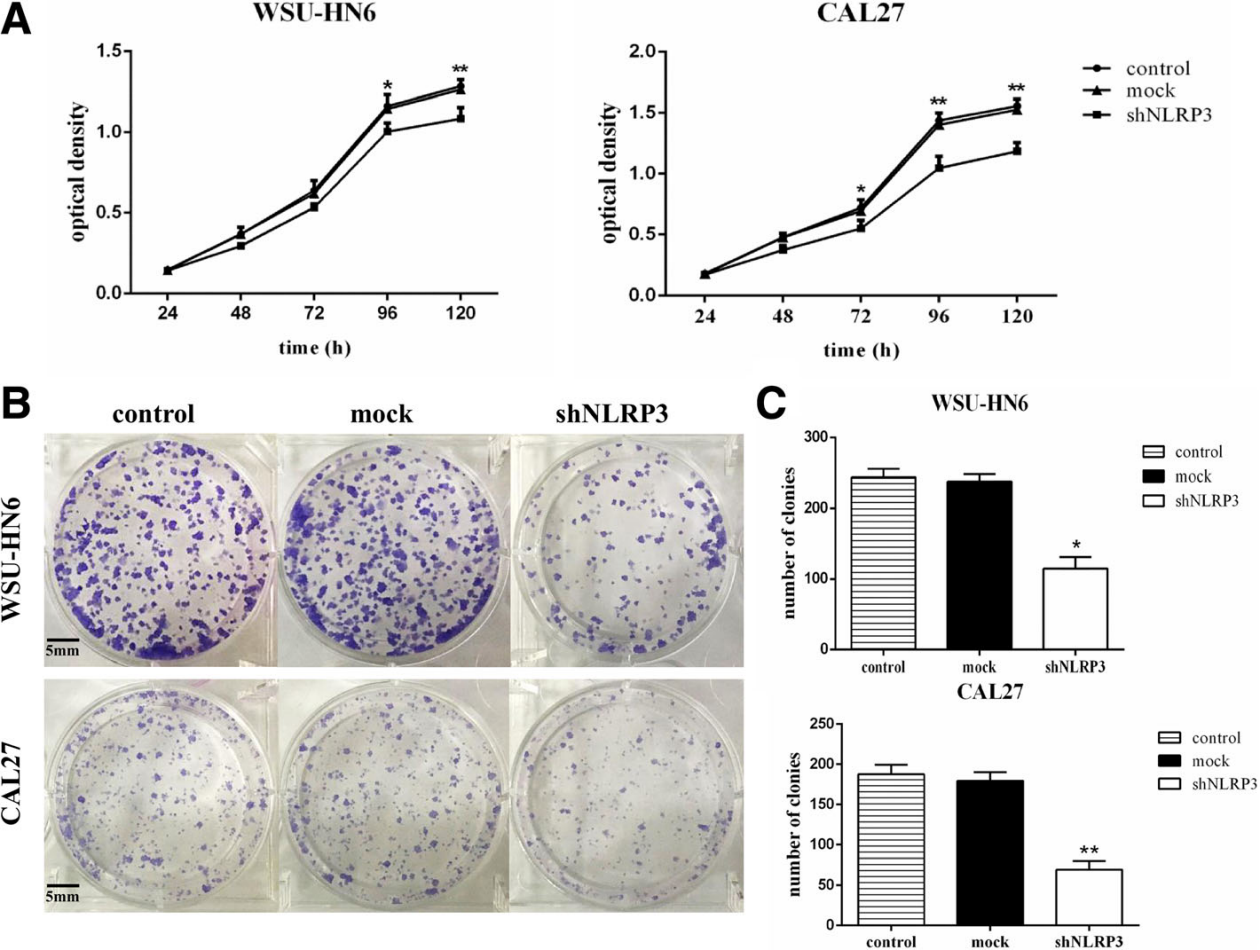
NLRP3 knockdown decreases OSCC cell proliferation. WSU-HN6 and CAL27 cells were transfected with shNLRP3 or mock vector. **a** Cell proliferative abilities of the cells were examined by the MTT assay. **b** OSCC cells were seeded into 6-well plates at a density of 800 cells/well. After 2 weeks, the cell clones were stained and photographed. **c** Histograms demonstrated the numbers of colonies in each group. One representative experiment out of three is shown (*P < 0.05, **P < 0.01)

To clarify the biological significance of NLRP3, NLRP3 short hairpin RNA was constructed and transfected into the OSCC cells. As higher NLRP3 expression was found in WSU-HN6 and CAL27 cells, we chose them for subsequent studies. The interference efficiency was determined, and markedly reduced NLRP3 expression was observed in OSCC cells after shNRLP3 transfection (Fig. 3). Similar expression patterns were also found for cleaved caspase-1 and IL-1β, which indicated that the function of NLRP3 inflammasome was impaired by NLRP3 knockdown. Then we assessed the proliferative abilities of OSCC cells, and found NLRP3 knockdown significantly decreased the cell viabilities and affected the colony formation of OSCC cells (Fig. 4). Thus, these results suggest that NLRP3 exerts an important role in OSCC cell proliferation.

### Decreased NLRP3 expression attenuates OSCC cell migration and invasion

**Fig. 5:**
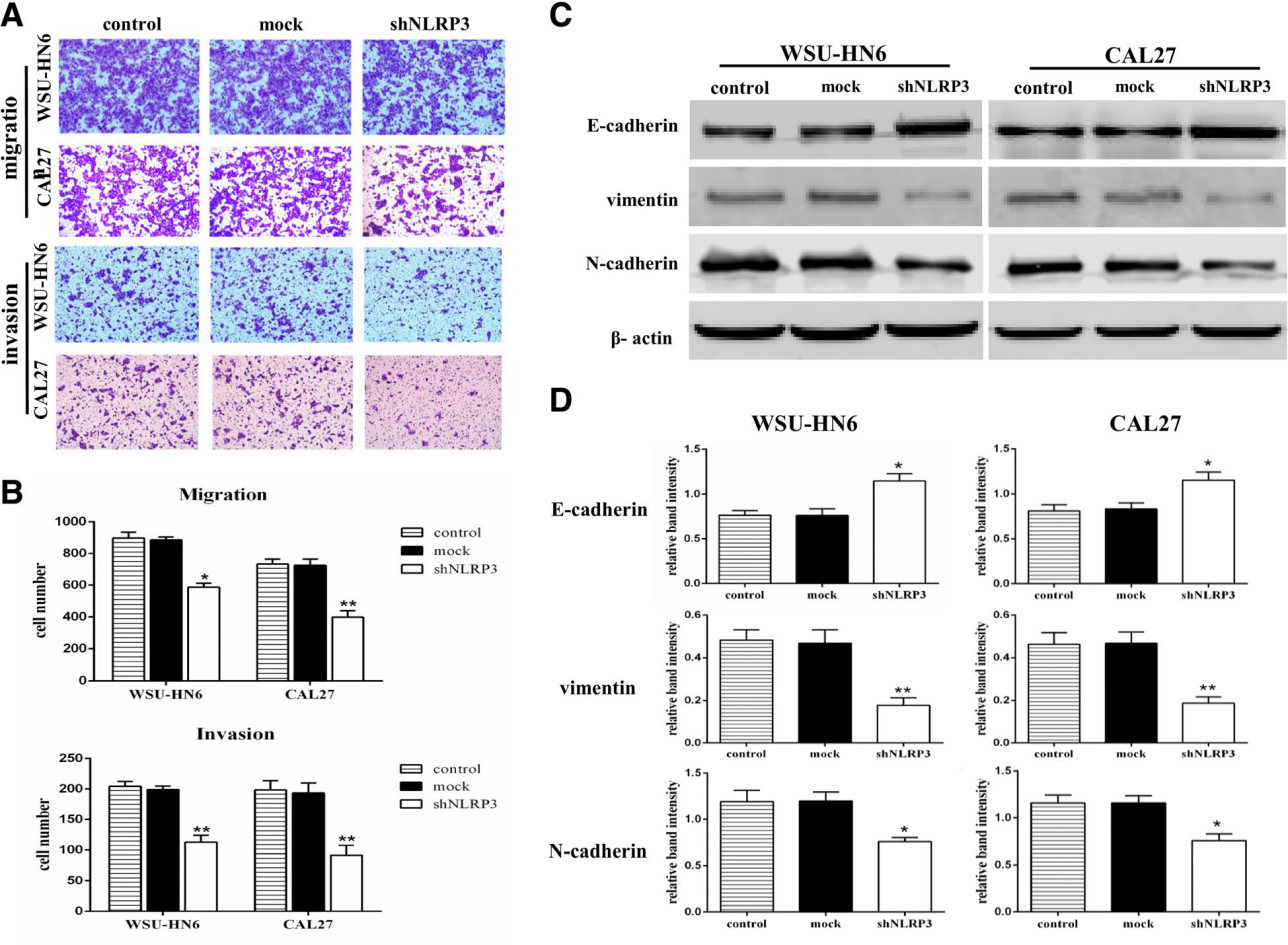
NLRP3 inhibition suppressed the migration and invasion of OSCC cells. **c** Representative images of the migration and invasion of OSCC cells with or without NLRP3 knockdown (magnification, 100×). **b** Quantitative data showed NLRP3 knockdown suppressed OSCC cell migration and invasion. **c** EMT-related proteins were determined by western blot. **d** The expression ratio of E-cadherin, vimentin and N-cadherin was quantified against β-actin. Results are representative of three experiments. (*P < 0.05, **P < 0.01)

Since NLRP3 expression was correlated with N stage which indicated the lymphonode metastasis status of OSCC, the effect of NLRP3 knockdown on OSCC cell migration and invasion was examined. Results showed that shNLRP3 transfection significantly decreased the migration and invasion of OSCC cells (Fig. 5a and b). Considering that the epithelial-mesenchymal transition (EMT) is a critical process in tumor metastasis, in which cancer cells lose epithelial morphology and acquire mesenchymal morphology and invasive phenotype, EMT-related proteins were determined. Consistently, the mesenchymal markers-vimentin and N-cadherin were clearly downregulated, while the epithelial adhesion marker E-cadherin was obviously upregulated in OSCC cells with shNLRP3 transfection (Fig. 5c and d). These data suggest that NLRP3 involves in the metastasis of OSCC cells.

### Suppression of NLRP3 affects OSCC growth in vivo

**Fig. 6:**
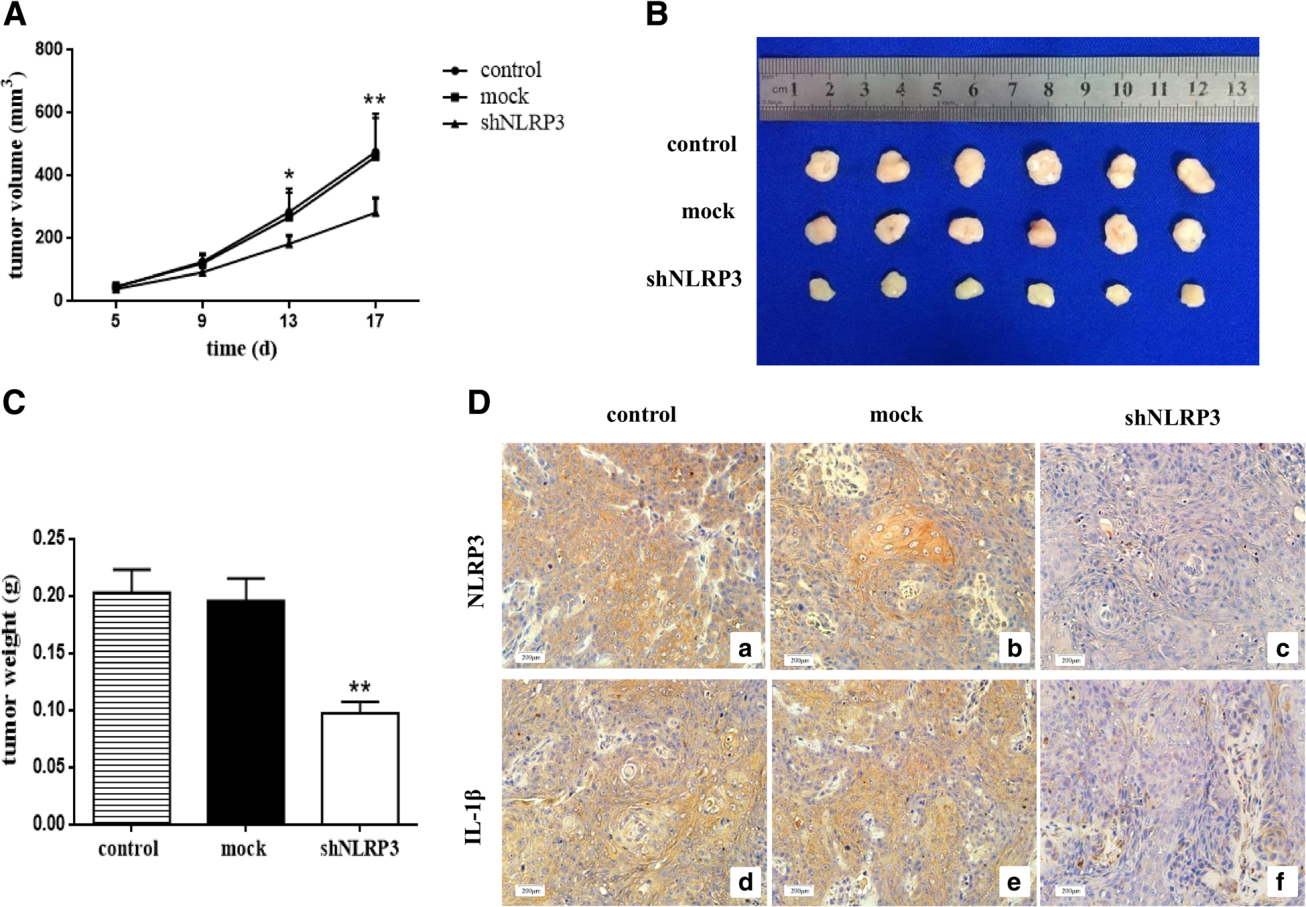
NLRP3 knockdown reduced OSCC cell growth in vivo. WSU-HN6 cells with or without NLRP3 knockdown were implanted into nude mice as described in Materials and methods. **a** Tumor volumes of each group at different time points. **b** Representative image of tumors derived from mice in each group. **c** Tumor weights of the three groups. **d** NLRP3 and IL-1β expression in tumors derived from parental cells, mock vector-transfected cells and shNLRP3-transfected cells were determined by IHC staining (original magnification 200×). Six mice were included for each group, and one representative experiment out of three is shown. (*P < 0.05, **P < 0.01)

To evaluate the effect of NLRP3 knockdown on the growth of OSCC cells in vivo, the OSCC xenograft model was established in nude mice. The parental cells (control), mock vector-transfected cells and shNLRP3-transfected cells (shNLRP3) were subcutaneously implanted into the back of the mice. Tumor volumes were calculated and tumor weights were recorded. Data demonstrated that the silencing of NLRP3 distinctly reduced the tumor sizes and weights (Fig. 6a-c). However, the growth of the mock-transfected tumor cells was not affected (Fig. 6a-c). Similar to the in vitro data, decreased expression of NLRP3 and IL-1β was found in tumors derived from shNLRP3-transfected cells compared with those from the control or mock-transfected cells (Fig. 6d). Therefore, NLRP3 silence impaired the function of NLRP3 inflammasome, which led to the suppressed OSCC growth in vivo.

## Discussion

OSCC is the most common head and neck squamous cell carcinoma, which can rise from various locations including the gingiva, tongue, floor of mouth and bucca [24]. The disease develops from precancerous dysplastic lesions through multistep processes of carcinogenesis and the related mechanisms were complex and poorly understood [25, 26]. In our present study, we demonstrated the OSCC-promoting property of NLRP3 both in vitro and in vivo for the first time. We found that NLRP3 was aberrantly overexpressed in OSCC cells, and the abnormal expression was correlated with the tumor stage, lymph node metastatic status and IL-1β expression level. Knockdown of NLRP3 significantly decreased OSCC cell proliferation, migration and invasion. Moreover, silencing NLRP3 expression significantly inhibited OSCC tumor growth in vivo.

Increasing evidences have emphasized the chronic inflammation is crucial for the initiation, growth and metastasis of cancer [27]. Therefore, the roles of the inflammation related immune molecules have received great attention. The pattern recognition receptors [PRRs, including the Toll-like receptors (TLRs) and NLRs etc.], which recognize the pathogen associated molecular pattern (PAMP) or damage associated molecular pattern (DAMP) and initiate the inflammatory responses, have been observed aberrantly expressed in various cancers [12]. Yang et al. reported that TLR4 was overexpressed in human breast cancer tissue and correlated with lymph node metastasis [28]. Zhou et al. found high expression of TLR5 correlated with better prognosis in non-small-cell lung cancer [29]. Moreover, TLR7 and TLR9 expression was discovered to be up-regulated in human hepatic cell carcinoma tissue [30]. For NLRs, the intensity of NLRP12 immunostaining was found significantly higher in malignant prostate as compared to their adjacent benign tissues [31]. Paugh et al. found NLRP3 inflammasome upregulation and caspase-1 cleavage of the glucocorticoid receptor caused glucocorticoid resistance in leukemia cells [22]. Our previous studies also reported the abnormal expression of PRRs-TLR3 and TLR4 in OSCC [32, 33]. In this study, we found NLRP3 was overexpressed in OSCC cells, and the expression was associated with the clinicopathological characteristics of the OSCC patients. Thus we inferred that the abnormal expression of NLRP3 in OSCC was functional and went on further investigation.

Previous studies indicated that the effects of NLRP3 inflammasome are complex with differential roles in different types of cancers. In the study of skin cancer, Chow et al. reported that NLRP3 promoted inflammation-induced skin cancer [34]. And in melanoma cells, Okamoto et al. found that overexpressed and constitutively activated NLRP3 inflammasome promoted late stage melanoma progression via the cleavaged caspase-1 and active IL-1β [16]. While in colitis-associated cancer, Allen et al. demonstrated that NLRP3 inflammasome in hematopoietic cells, rather than intestinal epithelial cells or stromal cells, functioned as a negative tumorigenesis regulator [35]. Moreover, chemotherapeutic agents may have an effect on the NLRP3 inflammasome of cells existed in the tumor microenvironment. Recently, Bruchard et al. reported that gemcitabine (Gem) and 5-fluorouracil (5-FU) activated the NLRP3 inflammasome in myeloid-derived suppressor cells and led to the secretion of IL-1β, and then the active IL-1β promoted the production of IL-17 by CD4^+^ T cells and finally blunted the anticancer efficacy [36]. Thus, in-depth understanding the expression and function of NLRP3 inflammasome may offer new insights into tumor therapy. Our data demonstrated the overexpression of NLRP3 in OSCC cells promoted OSCC tumor growth and metastasis. And 5-FU treatment could further increase the expression and activation of NLRP3 inflammasome in OSCC cells, which in turn attenuated its anti-tumor effect [23]. However, the activation and function of NLRP3 inflammasome in other cells of the OSCC tumor microenvironment needs more exploration.

## Conclusions

In conclusion, our results reveal that the overexpressed NLRP3 contributes to the proliferation and metastasis of OSCC cells. Thus, NLRP3 may be used as a potential target for OSCC treatment.

## Additional file

Additional file 1: **Table S1.** IHC Staining score of the expression levels of NLRP3 and IL-1β in 77 OSCC tissues. The positive grade was determined based on the Immuno-Reactive-Score (IRS), which was calculated by multiplying staining proportion score (PS) and staining intensity score (IS). PS was classified as 0 (0%), 1 (1%–25%), 2 (26%–50%), 3 (51%–75%), and 4 (76%–100%). IS was classified as 0 (negative), 1 (weak), 2 (moderate) and 3 (strong). The final staining score was calculated by multiplying IS by PS. According to the final score, patients with different expression were divided into three groups: negative expression group (IRS = 0), weak expression group (IRS = 1–3) and strong positive expression group (IRS = 4–12). (XLSX 15 kb)

## Abbreviations

5FU: 5-fluorouracil
AJCC: American Joint Committee on Cancer
ASC: Speck-like protein containing CARD
DAMP: Damage associated molecular pattern
DMEM: Dulbecco’s modified Eagle medium
DMSO: Dimethyl sulfoxide
EMT: Epithelial-to-mesenchymal transition
FBS: Fetal bovine serum
Gem: Gemcitabine
HIOEC: Human immortalized oral epithelial cells
IHC: Immunohistochemistry
IRS: Immuno-Reactive-Score
IS: Staining intensity score
MTT: 3-(4,5-dimethylthiazol-2-yl)-2,5-diphenyl tetrazolium bromide
NLR: Nucleotide-binding and oligomerization domain (NOD)-like receptor
NLRP3: NLR pyrin domain-containing protein 3
OSCC: Oral squamous cell carcinoma
PAMP: Pathogen associated molecular pattern
PBS: Phosphate-buffered saline
PS: Staining proportion score
PVDF: Polyvinylidene difluoride
RIPA: Radio immunoprecipitation assay
SDS: Sodium dodecyl sulfate
